# Supplementary material for: Attitudes and current practice in alcohol screening, brief intervention, and referral for treatment among staff working in urgent and emergency settings: An open, cross-sectional international survey
Source: PLoS One. 2023 Sep 27;18(9):e0291573. doi: 10.1371/journal.pone.0291573 (PMC10529549; doi:10.1371/journal.pone.0291573)
Supplement: S1 File — (PDF) [file pone.0291573.s002.pdf]

S2 Supporting Information
Supplementary file 2: Example survey invitation.

Text was adapted to suit the platform (e.g., social media, email). Example text:

Dear [e.g., professional network lead],

Would you be willing to circulate the email below around any relevant professional networks and groups (and social media)?

Do you work in urgent or emergency care?
What are your views about health promotion / alcohol prevention in emergency settings?
Will you help to inform future practice by sharing your views?

If so, click here [insert survey link]:

We are seeking the views of individuals who work in urgent or emergency care settings towards the delivery of health promotion, and specifically alcohol prevention, in these environments.

The survey is relevant to individuals from any occupational group and any geographical region.

By completing and submitting the survey you are giving written informed consent to participate. The study has research ethics approval from the University of [XXX] (Ref: FMHS 415-1121).

Many thanks,

[Chief Investigator name]

[University of…]
