# Supplementary material for: Attitudes and current practice in alcohol screening, brief intervention, and referral for treatment among staff working in urgent and emergency settings: An open, cross-sectional international survey
Source: PLoS One. 2023 Sep 27;18(9):e0291573. doi: 10.1371/journal.pone.0291573 (PMC10529549; doi:10.1371/journal.pone.0291573)
Supplement: S2 File — (PDF) [file pone.0291573.s003.pdf]

[redacted]

Alcohol prevention in urgent and emergency care

Showing 0 of 0 responses

Showing **all** responses

Showing **all** questions

1 Q1. What is your age?

16-20	0
21-30	0
31-40	0
41-50	0
51-65	0
66+	0

2 Q2. What gender do you identify as?

Male	0
Female	0
Non-binary/Gender fluid	0
Prefer not to disclose	0
Other	0

3 Q3. What is your ethnicity?

White - British	0
White - Irish	0
White - Other White background	0
Mixed – White and Black Caribbean	0
Mixed – White and Black African	0
Mixed – White and Asian	0
Mixed – Other Mixed background	0
Asian/Asian British – Indian	0
Asian/Asian British – Pakistani	0
Asian/Asian British – Bangladeshi	0
Asian/Asian British – Other Asian background	0
Black/Black British – Caribbean	0
Black/Black British – African	0
Black/Black British – Other Black background	0
Chinese and other ethnic background - Chinese	0
Chinese and other ethnic background - Other ethnic background	0

4 Q4. What is your highest qualification? (Select one)

Degree level or above	0
Another kind of qualification	0
No qualifications	0

5 Q5. What is your occupational group?

Paramedic	0
Emergency department (ED) physician	0
Urgent Care Physician / GP	0
Advanced Clinical Practitioner (ACP) – an advanced role, not limited to traditional boundaries of clinical specialisms.	0
Registered Nurse (RN)	0
Nurse Practitioner (NP)	0
Enhanced Clinical Practitioner (ECP) – an enhanced role, not limited to traditional boundaries of clinical specialisms.	0
Nursing/Healthcare Assistant	0
General Manager	0
Other (please specify	0

5.a If you selected Other, please specify:

No responses

6 Q6. How long have you worked in your current role?

Less than 1 year	0
1	0
2	0
3	0
4	0
5	0
6	0
7	0
8	0
9	0
10	0
11	0
12	0
13	0
14	0
15	0
16	0
17	0
18	0
19	0
20	0
21	0
22	0
23	0
24	0
25	0
26	0
27	0
28	0
29	0
30	0
31	0
32	0
33	0
34	0
35	0
35+	0

7 Q7. What is your setting?

Emergency Department	0
Urgent Care Centre	0
Minor Injury Unit	0
Drop-in Centre	0
Walk-in Centre	0
Other (Please specify)	0

7.a If you selected Other, please specify:

No responses

8 Q8. In which geographical region do you work?

UK	0
Europe	0
North America/Central America	0
South America	0
Africa	0
Asia	0
Australia	0
Caribbean Islands	0
Pacific Islands	0
Other	0

8.a If you selected Other, please specify:

No responses

8.b If selected UK, please specify which region;

England	0
Wales	0
Scotland	0
Northern Ireland	0

9 Q9. What is your work pattern?

Standard office hours (e.g., 9-5)	0
Shifts	0
Other	0

9.a If you selected Other, please specify:

No responses

10 Q10. Do you see health promotion as important?

very important	0
moderately important	0
not important	0

11 Q11. Do you see health promotion as part of your current professional role?

Yes	0
Unsure	0
No	0

12 Q12. Have you undertaken any training on delivering brief interventions for lifestyle behaviours?

Yes	0
No	0

12.a Q12a. If yes, describe the training;

No responses

13 Q13. Is there a need for SBIRT (screening, brief advice, referrals) for alcohol in urgent and emergency settings?

Yes	0
No	0

14 Q14. Do you think urgent and emergency settings are an appropriate place to deliver SBIRT (screening, brief advice, referrals) for alcohol?

14.1 Q14a. Screening

14.1.a Q14a. Screening

Yes	0
No	0

14.2 Q14b. Brief advice

14.2.a Q14b. Brief advice

Yes	0
No	0

14.3 Q14c. Referrals

14.3.a Q14c. Referrals

Yes	0
No	0

15 Q15. Do you think it is practical to implement SBIRT (screening, brief advice, referrals) in urgent and emergency settings?

Practical	0
Not sure / it depends	0
Impractical	0

16 Q16. Have you EVER delivered SBIRT (screening, brief advice, referrals) for alcohol?

Yes	0
No	0

16.a Was it Screening?

Yes	0
No	0

16.a.i Was it acceptable to patients?

Yes	0
No	0

16.b Was it Brief advice?

Yes	0
No	0

16.b.i Was it acceptable to patients?

Yes	0
No	0

16.c Was it Referrals?

Yes	0
No	0

16.c.i Was it acceptable to patients?

Yes	0
No	0

17 Q17. Do you CURRENTLY deliver SBIRT (screening, brief advice, referrals) for alcohol?

17.1 Q17a. Screening

17.1.a Q17a. Screening

Yes	0
No	0

17.2 Q17b. Brief advice

17.2.a Q17b. Brief advice

Yes	**0**
No	**0**

17.3 Q17c. Referrals

17.3.a Q17c. Referrals

Yes	**0**
No	**0**

18 Q18. Would you be willing to deliver SBIRT (screening, brief advice, referrals) for alcohol in the FUTURE?

Yes	**0**
No	**0**

19 Q19. Have you used any screening measures for alcohol use? (Please select all that apply)

Alcohol Use Disorders Identification Test (AUDIT)	0
Alcohol Use Disorders Identification Test for Primary Care (AUDIT-PC)	0
Alcohol Use Disorders Identification Test for Consumption (AUDIT-C)	0
Fast Alcohol Use Screening Test (FAST)	0
Modified Single Alcohol Screening Questionnaire (M-SASQ)	0
Paddington Alcohol Test (PAT)	0
None	0
Other	0

Multi answer: Percentage of respondents who selected each answer option (e.g. 100% would represent that all this question's respondents chose that option)

19.a If you selected Other, please specify:

No responses

20 Q20. Have you given a brief intervention (advice) for alcohol?

Yes	0
No	0

20.a Q20a. If yes, what method have you used? (Please select all that apply)

Verbal education or advice	0
Brochure/leaflet	0
Signpost to website	0
Other	0

Multi answer: Percentage of respondents who selected each answer option (e.g. 100% would represent that all this question's respondents chose that option)

20.a.i If you selected Other, please specify:

No responses

21 Q21. In your setting, what is current practice regarding referral to treatment with respect to alcohol? (Please select all that apply)

Psychological Treatment / Services (e.g., Psychotherapy, Cognitive Behavioural Therapy, Dialectical Behavioural Therapy, Behavioural Couples Therapy)	0
Specialist Alcohol Counselling	0
Brief Intervention (e.g., Motivational Interviewing, Solutions-Focused Approach)	0
12-Step Facilitation Programme (e.g., Alcoholics Anonymous)	0
Inpatient unit or a medically supported residential service	0
Intensive community rehabilitation programme	0
Social network and environment-based therapies	0
Lifestyle intervention (e.g., yoga, meditation)	0
Creative therapy (e.g., art and music therapy)	0
Other	0

Multi answer: Percentage of respondents who selected each answer option (e.g. 100% would represent that all this question's respondents chose that option)

21.a If you selected Other, please specify:

No responses

22 Q22. Have you ever made an alcohol referral yourself? (Please select all that apply)

Psychological Treatment / Services (e.g., Psychotherapy, Cognitive Behavioural Therapy, Dialectical Behavioural Therapy, Behavioural Couples Therapy)	0
Specialist Alcohol Counselling	0
Brief Intervention (e.g., Motivational Interviewing, Solutions-Focused Approach)	0
12-Step Facilitation Programme (e.g., Alcoholics Anonymous)	0
Inpatient unit or a medically supported residential service	0
Intensive community rehabilitation programme	0
Social network and environment-based therapies	0
Lifestyle intervention (e.g., yoga, meditation)	0
Creative therapy (e.g., art and music therapy)	0
Other	0

Multi answer: Percentage of respondents who selected each answer option (e.g. 100% would represent that all this question's respondents chose that option)

22.a If you selected Other, please specify:

No responses

23 Q23. To what extent do you agree with the following statements?

23.1 Q23a. Delivering SBIRT (alcohol screening, brief advice, and referral) will ultimately decrease urgent and emergency care attendance and hospitalisations.

23.1.a Q23a. Delivering SBIRT (alcohol screening, brief advice, and referral) will ultimately decrease urgent and emergency care attendance and hospitalisations.

Strongly agree	0
Agree	0
Disagree	0
Strongly disagree	0

23.2 Q23b. Reinforcing advice about alcohol use to patients in ED will prompt them to seek help.

23.2.a Q23b. Reinforcing advice about alcohol use to patients in ED will prompt them to seek help.

Strongly agree	0
Agree	0
Disagree	0
Strongly disagree	0

23.3 Q23c. Having conversations about alcohol with my patients is supported in my organisation.

23.3.a Q23c. Having conversations about alcohol with my patients is supported in my organisation.

Strongly agree	0
Agree	0
Disagree	0
Strongly disagree	0

23.4 Q23d. Having conversations about alcohol with my patients is supported by my colleagues.

23.4.a Q23d. Having conversations about alcohol with my patients is supported by my colleagues.

Strongly agree	0
Agree	0
Disagree	0
Strongly disagree	0

23.5 Q23e. My colleagues have conversations with their patients about alcohol.

23.5.a Q23e. My colleagues have conversations with their patients about alcohol.

Strongly agree	0
Agree	0
Disagree	0
Strongly disagree	0

23.6 Q23f. I understand the concept of a brief intervention for alcohol prevention.

23.6.a Q23f. I understand the concept of a brief intervention for alcohol prevention.

Strongly agree	0
Agree	0
Disagree	0
Strongly disagree	0

23.7 Q23g. I know how to screen patients for alcohol consumption

23.7.a Q23g. I know how to screen patients for alcohol consumption

Strongly agree	0
Agree	0
Disagree	0
Strongly disagree	0

23.8 Q23h. I know how to give brief advice to patients about reducing alcohol consumption.

23.8.a Q23h. I know how to give brief advice to patients about reducing alcohol consumption.

Strongly agree	0
Agree	0
Disagree	0
Strongly disagree	0

23.9 Q23i. I know how to make alcohol referrals.

23.9.a Q23i. I know how to make alcohol referrals.

Strongly agree	0
Agree	0
Disagree	0
Strongly disagree	0

23.10 Q23j. I have the skills to screen patients for alcohol consumption.

23.10.a Q23j. I have the skills to screen patients for alcohol consumption.

Strongly agree	0
Agree	0
Disagree	0
Strongly disagree	0

23.11 Q23k. I have the skills to give brief advice to patients about reducing alcohol consumption.

23.11.a Q23k. I have the skills to give brief advice to patients about reducing alcohol consumption.

Strongly agree	0
Agree	0
Disagree	0
Strongly disagree	0

23.12 Q23l. I have the skills to make alcohol referrals.

23.12.a Q23l. I have the skills to make alcohol referrals.

Strongly agree	0
Agree	0
Disagree	0
Strongly disagree	0

23.13 Q23m. I am confident in my ability to screen patients for alcohol consumption.

23.13.a Q23m. I am confident in my ability to screen patients for alcohol consumption.

Strongly agree	0
Agree	0
Disagree	0
Strongly disagree	0

23.14 Q23n. I am confident in my ability to have a conversation with patients about reducing alcohol consumption.

23.14.a Q23n. I am confident in my ability to have a conversation with patients about reducing alcohol consumption.

Strongly agree	0
Agree	0
Disagree	0
Strongly disagree	0

23.15 Q23o. I am confident in my ability to making alcohol referrals.

23.15.a Q23o. I am confident in my ability to making alcohol referrals.

Strongly agree	0
Agree	0
Disagree	0
Strongly disagree	0

23.16 Q23p. I have access to the resources and information I need to discuss alcohol with patients.

23.16.a Q23p. I have access to the resources and information I need to discuss alcohol with patients.

Strongly agree	0
Agree	0
Disagree	0
Strongly disagree	0

24 Q24. What are the key barriers to delivering SBIRT (screening, brief advice, referrals for alcohol) with patients in urgent and emergency settings? (Please select all that apply)

24.1 Q24a. Lack of training

24.1.a Q24a. Lack of training

Yes	0
No	0

24.1.b Q24a. Lack of training - If Yes, please specify which parts of SBIRT (please select all that apply)

Screening	0
Brief intervention	0
Referral to treatment	0

Multi answer: Percentage of respondents who selected each answer option (e.g. 100% would represent that all this question's respondents chose that option)

24.2 Q24b. Lack of knowledge on which patients are suitable

24.2.a Q24b. Lack of knowledge on which patients are suitable

Yes	0
No	0

24.2.b Q24b. Lack of knowledge on which patients are suitable - If Yes, please specify which parts of SBIRT (please select all that apply)

Screening	0
Brief intervention	0
Referral to treatment	0

Multi answer: Percentage of respondents who selected each answer option (e.g. 100% would represent that all this question's respondents chose that option)

24.3 Q24c. Lack of knowledge on the process

24.3.a Q24c. Lack of knowledge on the process

Yes	0
No	0

24.3.b Q24c. Lack of knowledge on the process - If Yes, please specify which parts of SBIRT (please select all that apply)

Screening	0
Brief intervention	0
Referral to treatment	0

Multi answer: Percentage of respondents who selected each answer option (e.g. 100% would represent that all this question's respondents chose that option)

24.4 Q24d. Lack of knowledge on how to start a conversation with a patient

24.4.a Q24d. Lack of knowledge on how to start a conversation with a patient

Yes	0
No	0

24.4.b Q24d. Lack of knowledge on how to start a conversation with a patient - If Yes, please specify which parts of SBIRT (please select all that apply)

Screening	0
Brief intervention	0
Referral to treatment	0

Multi answer: Percentage of respondents who selected each answer option (e.g. 100% would represent that all this question's respondents chose that option)

24.5 Q24e. Lack of knowledge about the effectiveness

24.5.a Q24e. Lack of knowledge about the effectiveness

Yes	0
No	0

24.5.b Q24e. Lack of knowledge about the effectiveness - If Yes, please specify which parts of SBIRT (please select all that apply)

Screening	0
Brief intervention	0
Referral to treatment	0

Multi answer: Percentage of respondents who selected each answer option (e.g. 100% would represent that all this question's respondents chose that option)

24.6 Q24f. Not enough time / workload is too heavy

24.6.a Q24f. Not enough time / workload is too heavy

Yes	0
No	0

24.6.b Q24f. Not enough time / workload is too heavy - If Yes, please specify which parts of SBIRT (please select all that apply)

Screening	0
Brief intervention	0
Referral to treatment	0

Multi answer: Percentage of respondents who selected each answer option (e.g. 100% would represent that all this question's respondents chose that option)

24.7 Q24g. Lack of personal interest

24.7.a Q24g. Lack of personal interest

Yes	0
No	0

24.7.b Q24g. Lack of personal interest - If Yes, please specify which parts of SBIRT (please select all that apply)

Screening	0
Brief intervention	0
Referral to treatment	0

Multi answer: Percentage of respondents who selected each answer option (e.g. 100% would represent that all this question's respondents chose that option)

24.8 Q24h. Lack of reimbursement

24.8.a Q24h. Lack of reimbursement

Yes	0
No	0

24.8.b Q24h. Lack of reimbursement - If Yes, please specify which parts of SBIRT (please select all that apply)

Screening	0
Brief intervention	0
Referral to treatment	0

Multi answer: Percentage of respondents who selected each answer option (e.g. 100% would represent that all this question's respondents chose that option)

24.9 Q24i. Expected patient denial of alcohol issue

24.9.a Q24i. Expected patient denial of alcohol issue

Yes	0
No	0

24.9.b Q24i. Expected patient denial of alcohol issue - If Yes, please specify which parts of SBIRT (please select all that apply)

Screening	0
Brief intervention	0
Referral to treatment	0

Multi answer: Percentage of respondents who selected each answer option (e.g. 100% would represent that all this question's respondents chose that option)

24.10 Q24j. Expected patient resistance to advice

24.10.a Q24j. Expected patient resistance to advice

Yes	0
No	0

24.10.b Q24j. Expected patient resistance to advice - If Yes, please specify which parts of SBIRT (please select all that apply)

Screening	0
Brief intervention	0
Referral to treatment	0

Multi answer: Percentage of respondents who selected each answer option (e.g. 100% would represent that all this question's respondents chose that option)

24.11 Q24k. Lack of high-quality information and resources

24.11.a Q24k. Lack of high-quality information and resources

Yes	0
No	0

24.11.b Q24k. Lack of high-quality information and resources - If Yes, please specify which parts of SBIRT (please select all that apply)

Screening	0
Brief intervention	0
Referral to treatment	0

Multi answer: Percentage of respondents who selected each answer option (e.g. 100% would represent that all this question's respondents chose that option)

24.12 Q24l. Lack of clinical pathways

24.12.a Q24l. Lack of clinical pathways

Yes	0
No	0

24.12.b Q24l. Lack of clinical pathways - If Yes, please specify which parts of SBIRT (please select all that apply)

Screening	0
Brief intervention	0
Referral to treatment	0

Multi answer: Percentage of respondents who selected each answer option (e.g. 100% would represent that all this question's respondents chose that option)

24.13 Q24m. My own alcohol consumption affects my willingness to engage

24.13.a Q24m. My own alcohol consumption affects my willingness to engage

Yes	0
No	0

24.13.b Q24m. My own alcohol consumption affects my willingness to engage - If Yes, please specify which parts of SBIRT (please select all that apply)

Screening	0
Brief intervention	0
Referral to treatment	0

Multi answer: Percentage of respondents who selected each answer option (e.g. 100% would represent that all this question's respondents chose that option)

24.a Are there any other barriers to SBIRT? Please specify;

No responses

25 Q25. What would help increase delivery of SBIRT (screening, brief advice, referrals) for alcohol by staff in urgent and emergency settings?

No responses

26 Q26. What are your training needs? (Please select all that apply)

Medical complications of alcohol use	0
Social and psychiatric problems faced by people with alcohol use disorders	0
Screening and early identification of alcohol use disorders	0
Techniques for delivery of brief interventions (having conversations about alcohol use)	0
Treatment options for people with alcohol use disorders (or alcohol-related problems)	0
Diagnosing and treating alcohol withdrawal	0
Alcohol abstinence / reduction strategies	0
Counselling strategies to increase patients' motivation to cut down/abstain	0
Making referrals to relevant services	0
None	0
Other	0

Multi answer: Percentage of respondents who selected each answer option (e.g. 100% would represent that all this question's respondents chose that option)

If you selected Other, please specify:

26.a

No responses

27 Q27. Is there a role for promoting the areas listed below in urgent and emergency settings? (Please select all that apply)

Physical activity	0
Diet / nutrition	0
Weight management / BMI	0
Health screening	0
Vaccination uptake	0
Other	0

Multi answer: Percentage of respondents who selected each answer option (e.g. 100% would represent that all this question's respondents chose that option)

27.a If you selected Other, please specify:

No responses

28 Q28. How easily could you introduce other elements of health promotion into your professional role in an urgent or emergency setting? 1 = extremely easy, through to 10 = extremely difficult

28.1 Physical activity

1	0
2	0
3	0
4	0
5	0
6	0
7	0
8	0
9	0
10	0

Multi answer: Percentage of respondents who selected each answer option (e.g. 100% would represent that all this question's respondents chose that option)

28.2 Diet / nutrition

1	0
2	0
3	0
4	0
5	0
6	0
7	0
8	0
9	0
10	0

Multi answer: Percentage of respondents who selected each answer option (e.g. 100% would represent that all this question's respondents chose that option)

28.3 Weight management / BMI

1	0
2	0
3	0
4	0
5	0
6	0
7	0
8	0
9	0
10	0

Multi answer: Percentage of respondents who selected each answer option (e.g. 100% would represent that all this question's respondents chose that option)

28.4 Health screening

1	**0**
2	**0**
3	**0**
4	**0**
5	**0**
6	**0**
7	**0**
8	**0**
9	**0**
10	**0**

Multi answer: Percentage of respondents who selected each answer option (e.g. 100% would represent that all this question's respondents chose that option)

28.5 Vaccination uptake

1	**0**
2	**0**
3	**0**
4	**0**
5	**0**
6	**0**
7	**0**
8	**0**
9	**0**
10	**0**

Multi answer: Percentage of respondents who selected each answer option (e.g. 100% would represent that all this question's respondents chose that option)

28.6 Other (please specify below)

1	0
2	0
3	0
4	0
5	0
6	0
7	0
8	0
9	0
10	0

Multi answer: Percentage of respondents who selected each answer option (e.g. 100% would represent that all this question's respondents chose that option)

28.a If you select Other, please specify;

No responses

29 Q29. If you would be happy to be contacted for future research, please provide a valid email address. Your contact details will be separated from your survey responses.

No responses
