# Supplementary material for: Attitudes and current practice in alcohol screening, brief intervention, and referral for treatment among staff working in urgent and emergency settings: An open, cross-sectional international survey
Source: PLoS One. 2023 Sep 27;18(9):e0291573. doi: 10.1371/journal.pone.0291573 (PMC10529549; doi:10.1371/journal.pone.0291573)
Supplement: Attachment — Submitted filename: Alcohol SBIRT_PONE.docx [file pone.0291573.s004.docx]

In the submitted manuscript titled: **Attitudes and current practice in alcohol screening, brief intervention, and referral for treatment among staff working in urgent and emergency settings: a survey-title may need to be modified to reflect study objectives.**

I would like to commend the authors for the research efforts in addressing a topic of great public health importance. Your study explores the subject of (SBIRT) for alcohol among professionals working in urgent and emergency care (UEC) settings, which is undoubtedly of significant interest.

While I appreciate the insights provided by your descriptive findings, I must express some reservations regarding the external validity of the study design. The limitations in the design might make it challenging to generalize the findings to broader populations or settings. Consequently, the extent to which these findings contribute to the existing body of knowledge on the views and experiences related to SBIRT in UEC settings may be limited.

In light of this, I would like to suggest that you reconsider your research objectives and consider analyzing additional information that could enhance the value of your study. By incorporating additional variables or employing alternative methodologies, you may be able to provide novel insights and augment the existing understanding of this subject matter.

Once again, I commend you for your valuable contributions to the field and encourage you to refine and expand upon your work to further advance knowledge in this area. I believe that by addressing the aforementioned concerns, your research has the potential to make a substantial impact on the scientific community and inform future studies.

Below are additional points that need to be clarified

1. **Title and abstract:**
i. It is important that the study design appears in the title
ii. Background seems to state the overall objective, you may consider modifying it to briefly discuss the topic under research.
iii. The methodology appears somehow redundant, needs to capture more important details such as how data were analyzed, statistical tests performed etc, explain briefly that you collected qualitative and quantitative data
iv. Discussion should not be part of an abstract
2. **Introduction:** Background information and rationale to the problem under study has been clearly stipulated however the objectives/study aim has not been stated here but rather in the methods, I would recommend moving the study aim from the methods to the introduction (see the STROBE Checklist)
3. **Methods**
i. Study aim: should move to the introduction section
ii. Study design: Adequate information on the study design has been provided.
iii. Questionnaire items: (line 133-142) you seem to have five objectives however the section mentioned in the tool seems not to capture all study objectives example, training needs to support future SBIRT practice.
iv. The study procedure has been comprehensively described however; the authors do not explain the intended sample size prior to data collection that would also determine when the data collection should have been stopped.
v. The ethical consideration has been explicitly stated.
4. **Results**
i. Table 1; I am not sure of the rationale for including age group 16-20 as it has no any participants
ii. Table 2; You could add a column that shows proportion out of the total (all cadres) to give an overall picture with regards to UEC
iii. P values have been presented, line 250, 251 and 262 however they do not seem to appear in the tables, kindly consider presenting them in the tables of results as well.
iv. Line 324; “the need to improve SBIRT delivery in UEC” should be numbered (i)
v. Qualitative findings have been well described however, missing adequate primary data to back up the messages/themes being portrayed. Kindly consider adding more quotes for transparency.
5. **Discussion**
i. The discussion is too long; needs to be cut down significantly with a focus on findings that answer study objectives
6. **Study limitations**
i. I recommend that the subtitle reads strengths and limitations rather than limitations alone.
ii. This section is also too long, needs to be focused; less emphasis & text on COVID-19 pandemic (lines 550-557).
iii. The authors should also consider providing additional study strengths and limitations
7. **Conclusion**
i. Line 572 may not be suitable for the conclusion but rather the study strengths above.
