# Supplementary material for: Attitudes and current practice in alcohol screening, brief intervention, and referral for treatment among staff working in urgent and emergency settings: An open, cross-sectional international survey
Source: PLoS One. 2023 Sep 27;18(9):e0291573. doi: 10.1371/journal.pone.0291573 (PMC10529549; doi:10.1371/journal.pone.0291573)
Supplement: Attachment — Submitted filename: Response to reviewers.docx [file pone.0291573.s005.docx]

**PONE-D-23-11156**

**Attitudes and current practice in alcohol screening, brief intervention, and referral for treatment among staff working in urgent and emergency settings: a survey.**

Dear PLOS ONE Editors,

Thank you for providing us with the opportunity to revise and resubmit this manuscript. We have endeavoured to address each comment below.

**Response to Editorial team:**

- We have updated the financial disclosure statement and provided this in the cover letter as requested.
- We do not have any figures in the manuscript (only tables and supplementary files).
- The grant information provided in the ‘Funding Information’ and ‘Financial Disclosure’ sections should now match.
- The grant number has now been added into the paper, cover letter statement, and in the online form.
- Author contributions have been checked and are accurate. Please note that none of the authors are employed by Nottingham Hospitals Charity (the funder). The online system would not recognise this funder name.
- The protocol is now published on protocols.io (dx.doi.org/10.17504/protocols.io.bp2l6xkn1lqe/v1) and the anonymized minimal dataset is available at The University of Nottingham Research Data Repository (doi: 10.17639/nott.7292). We have added a statement about this into the ‘ethics and consent to participation’ section, after the ethical approval reference. The data availability statement has been amended to include this information.
- Captions for Supporting Information files have been provided at the end of the manuscript and the in-text citations match accordingly.

**Response to reviewers:**

**1. Is the manuscript technically sound, and do the data support the conclusions?
The manuscript must describe a technically sound piece of scientific research with data that supports the conclusions. Experiments must have been conducted rigorously, with appropriate controls, replication, and sample sizes. The conclusions must be drawn appropriately based on the data presented.**

**Reviewer #1: Yes**

**Reviewer #2: Yes**

Thank you for this positive response.

**2. Has the statistical analysis been performed appropriately and rigorously?**

**Reviewer #1: Yes**

**Reviewer #2: Yes**

Thank you for this positive response.

**3. Have the authors made all data underlying the findings in their manuscript fully available?**

**Reviewer #1: Yes**

**Reviewer #2: No**

The protocol is published on protocols.io (doi: awaiting assignment) and the minimal dataset is available at The University of Nottingham Research Data Repository (doi: 10.17639/nott.7292).

**4. Is the manuscript presented in an intelligible fashion and written in standard English?

Reviewer #1: Yes**

**Reviewer #2: Yes**

Thank you for this positive response.

**Reviewer #1: The research topic being addressed is of great public health importance however, there are limitations in generalisability and knowledge that is generated from these findings. You may consider making revisions on the objectives and analysis so as to bring forth novel insights in the topic.**

Thank you for this suggestion. We have conducted further analyses to explore whether there are differences in variables (attitudes and views) between demographic groups, setting (UK or international) and occupations. This has been added to the results and reflected on in the discussion.

**Reviewer #2: I thank the authors for a well-structured and much needed evidence on the implementation of SBIRT in the urgent and emergency settings. The paper is well and extensively drafted, here are my comments to the authors.**

Thank you for this positive feedback.

**Abstracts
Line 37-38: The sentence is incomplete, consider revising.**

The sentence was in abbreviated language for the abstract but has now been expanded.

**Line 38-39: Authors indicated to involve “participants from any occupational group, working in urgent and emergency care (UEC) settings in any country or region”. Was this a multi-country study? I would advise indicating study setting in the abstract or title.**

This was an international survey and we have amended the title and abstract to reflect this. We have chosen not to use the term ‘multi-country’ as we did not specify or target specific countries so this term could be mis-interpreted.

**Line40 - 41: The sentence is incomplete, consider adding “we included 362 respondents (aged 21 - 65 years, 87.8% shift workers) from 7 occupational groups…”**

The sentence was in abbreviated form for the abstract but has now been expanded. We have chosen to use ‘There were…’ rather than ‘We included…’ to avoid any ambiguity.

**Line 48 – 53: To keep the abstract succinct, I would advise the authors to have four sections in the abstract – Background (Including objectives), Methods, Results, Conclusion**

Thank you for this suggestion, we have amended the abstract sub-headings.

**Background
Please add the study aim in the last paragraph of the background section.**

This has been actioned – aims and objectives have been moved to the end of the introduction.

**Method
Line 103 – 109: The authors should consider moving the study objective to the last paragraph of the background.**

This has been actioned – aims and objectives have been moved to the end of the introduction (and re-written for clarity).

**For clarity, I would advise the following flow for the method section
Study design and setting
Study population
Sampling and Sample size
Study procedures
Data Collection
Data Analysis
Ethical Considerations**

Thank you for the suggestion, this has been actioned.

**Line 161 – 163: Did the Authors mean “Participant information sheet included the purpose of the study…”**

Yes, the word ‘sheet’ has been added.

**Results
Table 1: Please summarise in the result section the variable “Target population” as documented for other variables’ findings.**

We have included the sub-heading ‘Study population’ (to align with sub-heading used previously in the methods).

**Table 1: The occupation group namely “linked profession” should be defined in the methods or table legend rather than defining it the results section.**

This is now included as a table legend as suggested:

^a^Linked profession: linked to / working directly with to ED patients, such as ED-based social prescribing; drug and alcohol liaison team; addiction psychiatrist; acute/critical care medicine physician.

**All tables should be referred in the text.**

All 5 tables are already referred to in the text.

**P values indicated in the results should be added in the tables.**

The only p values in the text related to a gender comparison which is not related to the content of the results table. Therefore, we prefer to leave this in the text.

**Line 212 – 217: Authors should avoid repeating what has been presented in the table.**

We have deleted this paragraph:

Physicians comprised almost half the study sample (n=176, 48.6%), with 146 (40%) from UK and 30 (8.3%) from overseas. Most respondents (n=318, 87.8%) reported working in shift patterns. Of shift workers, 74.1% (n=268) were from UK and 13.8% (n=50) were from overseas.

**Line 223: Consider removing the first statement. Line 242: The first statement should be removed. Line 259: The first statement should be removed.**

This statement has been removed from each section where it appeared.

**Line 315 – 316: Authors should ensure uniformity in presenting the results, all number should be accompanied by percentages.**

Thank you for the suggestion, we have included the missing percentages together with the denominator (those who responded to the training needs question item).

**Line 318: change the subtitle “Analysis of qualitative free text responses” to Qualitative findings.**

This has been actioned.

**Line 324: Change numbering from (ii) to (i)**
This has been actioned.

**Major comments
Line 151 – 154: It is not clear what link exists between COVID-19 pandemic and the rationale for the current study. Please elaborate.**

This is important context indicating that the survey was undertaken during a time of extreme stress and pressure for the health and care workforce, with evidenced workforce shortages and burnout – this could potentially influence staff views on the practicalities of undertaking prevention activities in the UEC environment during this high-pressured time.

The wording has been tightened up to reflect this.

**Line 154 – 155: The authors mentioned the use of convenient sampling in this study. I suggest having a separate section on sampling and sample size. Was a minimum sample size determined? Authors should justify the sample size decided for this study.**

Thank you for this suggestion. This has been included in a separate section in the methods ‘Sampling and sample size’.

**Line: For how long was the link posted in the social media platforms?**

Data were collected from March to December 2022 and reminders were posted at least weekly via different distribution channels (e.g., emails, social media). This information is included in the manuscript.

**Line 190 – 192: The authors mentioned the use of chi square in assessing the differences in views and practices relating to SBIRT according to demographic characteristics with statistical significance set at 5%. The findings depicting statistical significance in the differences in views should be indicated.**

We have significantly revised the results to provide more information and also results of new analyses requested by reviewers.

**Line 347 -: Authors should consider developing a different theme on the strength of SBIRT. The results presented here do not align with the theme “The need to improve SBIRT delivery in UEC”.**

We have re-considered the theme labels and have re-labelled theme (i) as ‘Barriers and enablers of SBIRT delivery’ which directly aligns to section content.

**Limitation: Since the authors could not report on non-response, authors should acknowledge the possibility of response bias**

We have added this to study limitations while recognising that efforts were made to minimise bias as described in study procedures.

**While revising your submission, please upload your figure files to the Preflight Analysis and Conversion Engine (PACE) digital diagnostic tool,** [**https://pacev2.apexcovantage.com/**](https://pacev2.apexcovantage.com/)**. PACE helps ensure that figures meet PLOS requirements. To use PACE, you must first register as a user. Registration is free. Then, login and navigate to the UPLOAD tab, where you will find detailed instructions on how to use the tool. If you encounter any issues or have any questions when using PACE, please email PLOS at** [**figures@plos.org**](mailto:figures@plos.org)**. Please note that Supporting Information files do not need this step.**

Not applicable. There are no figures in this paper.

**REVIEWER 2**

**In the submitted manuscript titled: Attitudes and current practice in alcohol screening, brief intervention, and referral for treatment among staff working in urgent and emergency settings: a survey-title may need to be modified to reflect study objectives.**

We feel the title reflects the study aims but have now modified the text to reflect the scope of the study, adding: ‘an international survey’.

**I would like to commend the authors for the research efforts in addressing a topic of great public health importance. Your study explores the subject of (SBIRT) for alcohol among professionals working in urgent and emergency care (UEC) settings, which is undoubtedly of significant interest.**

Thank you for this positive comment.

**While I appreciate the insights provided by your descriptive findings, I must express some reservations regarding the external validity of the study design. The limitations in the design might make it challenging to generalize the findings to broader populations or settings. Consequently, the extent to which these findings contribute to the existing body of knowledge on the views and experiences related to SBIRT in UEC settings may be limited.**

**In light of this, I would like to suggest that you reconsider your research objectives and consider analyzing additional information that could enhance the value of your study. By incorporating additional variables or employing alternative methodologies, you may be able to provide novel insights and augment the existing understanding of this subject matter.**

Thank you for your comment. Our study aims are objectives align with the proposal for which we received ethical approval, and the participants were provided with information that indicated our study focus. We therefore cannot add any additional variables or employ new methods.

However, to address concerns about the inclusion of only descriptive material (except for a small number of group comparisons that were included in the original manuscript), we have added some further analyses to compare attitudes and views according to demographic characteristics, setting (UK or international) and occupation. This has been added to the study objectives, and the findings are included in the results section and reflected on in the discussion. We believe this addresses your comment and adds value to the manuscript.

**Once again, I commend you for your valuable contributions to the field and encourage you to refine and expand upon your work to further advance knowledge in this area. I believe that by addressing the aforementioned concerns, your research has the potential to make a substantial impact on the scientific community and inform future studies.**

Thank you for this positive comment.

Below are additional points that need to be clarified

1. **Title and abstract:**
i. **It is important that the study design appears in the title**

We had previously included the word ‘survey’ in the title, this has now been expanded to ‘an open, cross-sectional international survey’.

ii. **Background seems to state the overall objective, you may consider modifying it to briefly discuss the topic under research.**

Thank you for this suggestion. The aims and objectives have been modified for clarity and better alignment with survey data collection sections, and sub-headings in the results.

iii. **The methodology appears somehow redundant, needs to capture more important details such as how data were analyzed, statistical tests performed etc, explain briefly that you collected qualitative and quantitative data**

We respectfully disagree that our methodology is redundant. To ensure the most important details were captured, we had carefully followed reporting guidelines in the “Strengthening the Reporting of Observational Studies in Epidemiology (STROBE) statement” and Checklist for Reporting Results of Internet E-Surveys (CHERRIES).

In our original manuscript, we had already specified that both quantitative and qualitative data were collected: “The survey collected quantitative data including responses on a Likert scale and categorical responses with non-response options (i.e., ‘not applicable’, ‘prefer not to say’), and qualitative data in free text responses”.

Further, there was already a section called ‘Data analysis’ which reports how our qualitative data from free text responses was analysed, how quantitative data are described, and which tests were performed on quantitative data.

However, we have now included more detail on the sampling strategy. We have also re-written the aims and objectives (as suggested elsewhere) which has made it clearer that we collected quantitative and qualitative data.

iv. **Discussion should not be part of an abstract**

Thank you for raising this, we have removed the word discussion and replaced it with conclusions.

2. **Introduction: Background information and rationale to the problem under study has been clearly stipulated however the objectives/study aim has not been stated here but rather in the methods, I would recommend moving the study aim from the methods to the introduction (see the STROBE Checklist)**

Thank you for this comment, we have moved the study aim to the introduction as suggested.

3. **Methods**
i. **Study aim: should move to the introduction section.**

This has been actioned.

ii. **Study design: Adequate information on the study design has been provided.**

Thank you for confirming.

iii. **Questionnaire items: (line 133-142) you seem to have five objectives however the section mentioned in the tool seems not to capture all study objectives example, training needs to support future SBIRT practice.**

The results directly map to study objectives and questionnaire items, although this was not clear enough. We have now improved the headings and heading levels to make this more obvious and added ‘training needs’ to section 5 of the survey items description. We believe this has improved the clarity.

iv. **The study procedure has been comprehensively described; however, the authors do not explain the intended sample size prior to data collection that would also determine when the data collection should have been stopped.**

We agree this was an important omission. This information has now been included in a separate section on sampling within the methods.

v. **The ethical consideration has been explicitly stated.**

Thank you for this positive comment.

4. **Results**
i. **Table 1; I am not sure of the rationale for including age group 16-20 as it has not any participants.**

We have deleted this row from the Table. Since this was a response option on the survey, we have added a Table footnote to ‘age’: ^a^There were no respondents in the 16-20 years category.

ii. **Table 2; You could add a column that shows proportion out of the total (all cadres) to give an overall picture with regards to UEC.**

Thank you for this suggestion, we have included a column showing the total and agree this helps with interpretation.

iii. **P values have been presented, line 250, 251 and 262 however they do not seem to appear in the tables, kindly consider presenting them in the tables of results as well.**

We have chosen to leave these in the text only, to avoid repetition, and because the gender comparison does not align with the content of the results table (Table 2).

iv. **Line 324; “the need to improve SBIRT delivery in UEC” should be numbered (i)**

This was a typographical error and has been corrected, thank you for picking this up.

v. **Qualitative findings have been well described however, missing adequate primary data to back up the messages/themes being portrayed. Kindly consider adding more quotes for transparency.**

Thank you for this suggestion. We have included many more quotes to illustrate the key messages and themes and believe this has improved the quality and flow of these sections.

5. **Discussion**
i. **The discussion is too long; needs to be cut down significantly with a focus on findings that answer study objectives.**

We have reviewed the discussion and respectfully prefer to retain the existing text as we cannot identify text that it would be helpful to remove.

The reason for this, is that we have discussed all our findings systematically. We have ensured that we have discussed findings related to each study objective, in the same order that the objectives and results are presented in methods and results. These findings are discussed in the context of literature and/or practice with suggestions for the future as would be expected in a discussion (e.g., what it means, and where next). We do not believe there is any information included that is not relevant to the study objectives.

Please note that the reviewer has also requested that we undertake further analyses as part of this revision process, and so there are now significantly more results and therefore more findings to reflect on in the discussion as we did identify some very interesting group differences. However, we have attempted to keep any new text as short as possible (a few sentences).

6. **Study limitations**
i. **I recommend that the subtitle reads strengths and limitations rather than limitations alone.**

This has been actioned.

ii. **This section is also too long, needs to be focused; less emphasis & text on COVID-19 pandemic (lines 550-557).**

The section is focused on any survey reporting criteria from the CHERRIES checklist that could not be met, as study limitations. Therefore, the text needs to remain to ensure we adhere to the checklist. We included contextual impacts on survey response, risk of bias, limitations of open surveys (no data on non-responders) and lack of cookies/IP addresses to determine unique visitors, data representativeness and limitations of free-text responses as qualitative data.

The pandemic is very important context here in terms of its potential influence on survey response, and on healthcare professional’s views as to whether health promotion (prevention) activities are plausible in UEC settings, due to its impact on clinical care, workforce shortages and stress levels. We would like to ensure this context remains in the section, but we have re-written the section to make the relevance clearer and removed any redundant wording. We have also removed reference to the pandemic from the conclusion.

iii. **The authors should also consider providing additional study strengths and limitations.**

Thank you for this suggestion, we have now amended this section (and moved line 572 from conclusions into the study strengths).

The section includes contextual impacts on survey response, risk of bias, limitations of open surveys (no data on non-responders) and lack of cookies/IP addresses to determine unique visitors, data representativeness and limitations of free-text responses as qualitative data.

7. **Conclusion**
i. **Line 572 may not be suitable for the conclusion but rather the study strengths above.**

Thank you for this suggestion, we have moved this from conclusion to study strengths and amended the subtitle to ‘Study strengths and limitations’.
