# Supplementary material for: Attitudes and current practice in alcohol screening, brief intervention, and referral for treatment among staff working in urgent and emergency settings: An open, cross-sectional international survey
Source: PLoS One. 2023 Sep 27;18(9):e0291573. doi: 10.1371/journal.pone.0291573 (PMC10529549; doi:10.1371/journal.pone.0291573)
Supplement: Attachment — Submitted filename: Response to Reviewers_third submission.docx [file pone.0291573.s006.docx]

Dear Editor,

Thank you for providing the opportunity to respond to the reviewer comments on the second submission. We have uploaded our third submission, in which we have endeavoured to respond to all comments either as a revision to the manuscript, or as a rebuttal.

**Reviewer 1.**

Thank you for confirming that:

No further revisions are required. All comments have been addressed. The manuscript is technically sound, and the data support the conclusions. The statistical analysis has been performed appropriately and rigorously, and the data have been made available. The manuscript is presented in an intelligible fashion and written in standard English.

**Reviewer 2.**

Thank you for confirming that:

No further revisions are required. All comments have been addressed. The manuscript is technically sound, and the data support the conclusions. The statistical analysis has been performed appropriately and rigorously, and the data have been made available. The manuscript is presented in an intelligible fashion and written in standard English.

**Reviewer 3.**

Thank you for confirming that:

The statistical analysis has been performed appropriately and rigorously, and the data have been made available. The manuscript is presented in an intelligible fashion and written in standard English.

Our response to the second set of comments is:

**This is a descriptive study using a modest convenience sample.**

This is accurate. As described in the methods, we adopted a convenience sampling strategy, targeting a sample size of 300-400 for a maximum sampling error of 5.0-5.8% [supported by reference 23].

With n=362 respondents, we therefore exceeded our minimum target sample size.

We are unable to change the study design but added further comparative analyses in response to the previous comments. In the discussion, we have reflected on the limitations of the sampling:

“The convenience sample approach may impact on external validity of the findings; some groups may be under- or over-represented. We have no data on non-responders and so cannot eliminate risk of response bias, although efforts to minimise bias are described in study procedures. The sample is not intended to be representative of all UEC settings globally but gives valuable and novel insights into the experiences and views of those in different regions and demonstrates that there are some differences in views and barriers to alcohol prevention between those working within the UK or elsewhere”.

Surveys with similar (or smaller) samples than ours have recently been published in PLOS ONE:

Schoultz M, McGrogan C, Beattie M, Macaden L, Carolan C, Dickens GL (2022) Uptake and effects of psychological first aid training for healthcare workers’ wellbeing in nursing homes: A UK national survey. PLoS ONE 17(11): e0277062. https://doi.org/10.1371/journal.pone.0277062

John-Henderson NA, Mueller CM (2020) The relationship between health mindsets and health protective behaviors: An exploratory investigation in a convenience sample of American Indian adults during the COVID-19 pandemic. PLoS ONE 15(11): e0242902. https://doi.org/10.1371/journal.pone.0242902

**At >9000 words, it is far too long. The authors did not take into account the previous reviewers comments about length. I would recommend reframing this paper as 1/2 brief reports.**

Thank you for your comment – we provide a rebuttal below.

The manuscript is not >9,000 words, the main text in our second submission was 8,145 words.

While this is significantly longer than our original submission, we were previously asked to add a large amount of new text (further detail required in every section), by three reviewers.

Reviewer 3 previously asked for more detail throughout the paper, and for all the results to be discussed in the context of the literature and in alignment with the study objectives. The reviewer also asked for further analyses in the results, these additional findings then also needed to be discussed. The reviewer requested previously that the qualitative section was expanded to include more quotes. We believe that we addressed all these requests for further detail, which naturally expanded the length – we tried to address requests as succinctly as possible and in the previous revision we removed some text from the limitations section.

We have ensured that all our findings are discussed systematically and in relation to each study objective. Our findings are discussed in the context of literature and/or practice with suggestions for the future as would be expected in a discussion (e.g., what it means, and where next).

We have also reported our study in line with two checklists and to adhere to this, specific information and statements needed to be included (CHERRIES checklist, STROBE statement).

Reviewer 3 has now presented a new request for findings to be discussed in the context of global literature (i.e., requiring additional text and context) but at the same time, has asked for the paper to be converted to a short report.

We have considered this request but feel that it would not be possible to convert the paper to a short report that is high quality, while also (i) adhering to the reporting guidelines we used, (ii) addressing comments from three reviewers that all involved requests for further detail, and (iii) including the additional global context which is a new request from Reviewer 3.

We do not believe it will add any value to split this paper into two. In our view, that would weaken the manuscript which currently benefits from the inclusion of both quantitative and qualitative data which is novel in this literature and findings have been triangulated in the interpretation and discussion.

It is important to note that two reviewers have already indicated that the additional text provided in the previous submission is satisfactory, and therefore removal of a significant amount of content at this stage would ‘undo’ the revisions made previously in response to all three reviewers.

Finally, the PLOS ONE guidelines clearly state:

“Manuscripts can be any length. There are no restrictions on word count, number of figures, or amount of supporting information”.

**The findings are not generalizable and do not offer any new insights into the challenges of implementing SBIRT for alcohol concerns into ED and other urgent care settings.**

We respectfully disagree that the manuscript does not offer any new insights.

New insights are:

- Evidence synthesis shows there is very little data from the UK and none from England. Only one survey has been done before that included UK participants and that only included Scotland. Most of our respondents were from the UK, including England, Ireland, Scotland. Therefore, we add new data to the literature on challenges of implementing SBIRT for alcohol concerns into ED and other urgent care settings, from regions not previously explored.
- Evidence synthesis has called for more research in general to establish whether incorporating health promotion into the roles of staff in UEC settings is acceptable – therefore there is a view that our knowledge in this field is limited. We have responded to this call for more research in this field.
- Our study includes facilitators to health promotion in UEC settings which is not well captured in prior research (as evidenced in the literature). This information is needed to help us move forwards from barriers/challenges to enabling.
- We gathered views of personnel working in a diverse range of UEC settings, across geographical regions (within and outside of the UK), varying in gender, age, and years of experience. This has not been done previously.
- The study includes both quantitative and qualitative responses which strengthens and contextualises findings – prior studies have not done this.
- The global context has significantly changed in recent years (given the impacts of the COVID-19 pandemic) and this study presents current views, based on the current healthcare context. This is also relevant with the increasing burden on healthcare services and the increasing need (or pressures) for embedding prevention activity across a range of contexts and settings. We demonstrate that UEC workers are willing to engage in prevention but experience barriers to doing so, they suggest factors which facilitate prevention such as training, and our findings demonstrate the facilitators are largely not in place (e.g., few staff are being trained in SBIRT despite being willing to do it). This leads to recommendations.

Regarding generalisability, we previously included this text in the limitations:

“The sample is not intended to be representative of all UEC settings globally but gives valuable and novel insights into the experiences and views of those in different regions and demonstrates that there are some differences in views and barriers to alcohol prevention between those working within the UK or elsewhere”.

**The study and findings are not adequately contextualised within the global literature.**

Thank you for this valuable comment.

We have already set our rationale in the context of global views towards health promotion and referred to research conducted across multiple countries in both the introduction and discussion, also using the terms ‘internationally’, ‘worldwide’, ‘global’ (etc) throughout our discussion.

However, we agree that more could be done to set the study findings in a global context with relation to health promotion policy, and ED attendance prevalence.

In the discussion, we have now added some context with relation to global policies (e.g., UK, Europe, US) and ensured that figures relating to alcohol-related attendances refer to variation across countries, with examples from UK, Europe, Australia and New Zealand, US.

We have added a brief sentence to clarify where research is either unavailable in other geographical regions and/or may be needed.

We have added 7 new references to support the global context but have kept the additional text very succinct given this reviewer’s concerns about manuscript length.
