# Supplementary material for: Attitudes and current practice in alcohol screening, brief intervention, and referral for treatment among staff working in urgent and emergency settings: An open, cross-sectional international survey
Source: PLoS One. 2023 Sep 27;18(9):e0291573. doi: 10.1371/journal.pone.0291573 (PMC10529549; doi:10.1371/journal.pone.0291573)
Supplement: Attachment — Submitted filename: Response to Editor.docx [file pone.0291573.s007.docx]

**Response to Editor**

Dear Editor,

Thank you for the opportunity to complete minor revisions on our manuscript.

**Journal requirements: Please review your reference list to ensure that it is complete and correct. If you have cited papers that have been retracted, please include the rationale for doing so in the manuscript text, or remove these references and replace them with relevant current references. Any changes to the reference list should be mentioned in the rebuttal letter that accompanies your revised manuscript. If you need to cite a retracted article, indicate the article’s retracted status in the References list and also include a citation and full reference for the retraction notice.**

We have reviewed the reference list and it is correct and aligns with author guidelines.

**Additional Editor Comments: Thanks for sharing the revised draft. Thanks for your responses to the raised comments. I agree that the manuscript is extra-long, which is acceptable for the PLOS journal. It would be helpful to reduce the number of references - 103. I suggest you reduced the references to maximum of 50. Also helpful to check all the references are in the correct format.**

We have removed 40 references and adjusted the text accordingly. This has significantly reduced the number of references in this paper (and removed some of the text).

Having reviewed the manuscript again, we believe that this is the maximum number we can remove without conducting a major re-write of the manuscript, and to ensure that we still (1) adhere to 2 sets of guidelines (CHERRIES and STROBE), and importantly, (2) retain all of the revisions made previously in response to 3 reviewers, over two rounds of review.

We hope that this is acceptable to the Editorial team.

We have checked the formatting of the references and ensured it is correct.

**Please proofread the whole document and be consistent in reporting (n,%) - check page 10, line 216. Be consistent with the reporting of p values - use 3 decimal points. Use one decimal point for proportions.**

Thank you for raising this. We have reviewed the whole manuscript carefully and ensured that the reporting is consistent.
